# Comparing the effectiveness of mindfulness and emotion regulation training in reduction of marital conflicts 

**Published:** 2015

**Authors:** H Molajafar, SM Mousavi, R Lotfi, Seyedeh Madineh Ghasemnejad, M Falah

**Affiliations:** * Islamic Azad University, Karaj Branch, Karaj, Iran; ** Department of Psychology, Rasht Branch, Islamic Azad University, Rasht, Iran; *** Gilan University of Medical Sciences, Rasht, Iran; **** Department of Nursing and Midwifery, Lahijab Branch, Islamic Azad University, Lahjan, Iran; ***** Nursing, Mazandaran University of Medical sciences, Ramsar, Iran

**Keywords:** marital conflicts, emotion regulation, mindfulness

## Abstract

**Introduction:**this study aimed to compare the effectiveness of mindfulness and emotion regulation training in the reduction of marital conflicts.

**Methodology:**the present evaluation was a quasi-experimental study with a pretest-posttest design and a control group. The population consisted of all clients who referred to Moein Counseling Center in Alborz province (Spring 2014) due to marital problems. Using the simple random sampling method, 45 married people were selected as the sample and divided into two experimental groups (15 participants in each) and a control group (15 participants). Mindfulness training sessions were held for the first experimental group and emotion regulation training sessions were held for the second experimental group while, the participants in the control group did not receive any training. The Marital Conflicts Questionnaire was used for data collection and the obtained data were analyzed through descriptive statistics and analysis of covariance.

**Results: **the results confirmed the main hypothesis of this study regarding the effectiveness of mindfulness and emotion regulation training in reduction of marital conflicts (p<0.001, F=43.41).

**Discussion and conclusion:** there was a significant difference between mindfulness training and emotion regulation training in the reduction of marital conflicts; thus, compared to the mindfulness training, emotion regulation training can be considered a more effective treatment of marital conflicts.

## Introduction

Conflicts and misunderstandings are very common in all families. However, sometimes, these encounters may lead to severe challenges. Today, families are involved in different types of conflicts and intense disputes leading to the distortion of the family as an institution in which the creation of healthy personalities is the main focus. Conflict can be defined as disagreement or incompatibility between people due to different objectives, perceptions, and interests [**4**]. Marital conflicts are kind of ongoing significant mismatches between married couples who are shown by at least one of them. “Significant” in this definition connotes the effects of the conflicts on married couples’ performance and “ongoing” signifies the conflicts, which will not disappear over time. Conflicting couples are disturbed by their partners’ habits and personality and normally have communication problems in different areas and difficulties in accepting each other’s differences [**26**]. Couples in modern societies face several problems in establishing intimate relationships. It is obvious that deficiencies in the emotional quality of the marriage, along with several other factors, adversely affect the couple’s lives. Deriving from the negative responses to the partner’s differences, marital conflicts are invigorated when feelings of anger, hostility, revenge, hatred, and jealousy or verbal and physical abuses dominate the couples’ relationships [**9**]. Oliver and Miller [**22**] showed that many of the conflicting couples continue their marriage successfully because they believe that their marital problems are sexual, financial, or even due to their relatives’ interference while, the origin of their problems is actually the absence of healthy emotional behaviors. Marital confusion and conflicts may cause attachment problems for the couples and lead to reduced happiness, life satisfaction, and self-esteem; they also increase symptoms of psychological distress. It has also been confirmed that the biggest predictor of marital dissatisfaction and divorce is neither financial problems nor lack of sexual attraction or love but the method by which couples manage their marital conflicts in challenging situations [**16**].

Maladaptive emotion regulation can lead to creation or expansion of the conflicts. When people consistently suppress their emotions or react impulsively, they will experience more negative emotions, less positive emotions, relationship problems and reduced quality life [**13**]. Emotion regulation consists of all conscious and non-conscious strategies used to increase, maintain, or decrease emotional, behavioral, and cognitive components of an emotional response to a challenging situation [**14**]. It also refers to the ability to understand and express emotions appropriately [**12**]. Emotion regulation training means teaching techniques to control/ reduce negative emotions and increase/ use positive emotions. Regarding the importance of emotion regulation in relationships, the results have indicated that negative emotion regulation strategies can predict negative emotions and low satisfaction while positive emotion regulation strategies can predict less negative emotions in relationships [**21**]. Moreover, it has been specified that emotion regulation strategies are associated with reduced negative emotions and conflicts and focus on positive emotion regulation strategies can enhance people’s understanding of emotion management in relationships [**10**]. Essentially, emotion regulation and its training have been considered important factors in the improvement of the couples’ relationships as well as their lives’ quality and satisfaction [**11**].

Based on previous studies, emotion regulation refers to the process by which people experience and express their emotions [**23**,**24**] Difficulties in emotion regulation may be due to lack of emotion regulation capability [**15**].

According to the emotion regulation model, emotion regulation is a unique process for the modification of emotional experiences in order to achieve social acceptance and be in an appropriate physical and emotional state to respond properly to both internal and external demands. Emotion regulation also refers to “regulation and adjustment” of emotional processes applied in adaptive performances; therefore, emotional irregularity refers to a process that ultimately disrupts adaptive performances [**18**]. In a study, Beck [**7**] found that control and regulation of emotions along with people’s self-awareness of their emotions significantly enhance their marital relationships. In another study [**8**], it was found that people who had participated in emotion regulation group therapy sessions had a better control over their communication and mood swings.

One way to prevent behavioral and communicational problems is the improvement of the couples’ mental capacity that can be done through mindfulness training [**2**,**5**]. Mindfulness-based interventions are considered as one of the third-generation or third-wave cognitive-behavioral therapies. Mindfulness is a type of meditation rooted in Eastern rituals and wisdom, particularly the teachings of Buddha [**25**]. Mindfulness training can be introduced as a simple way to train the mind to be in a flexible and adaptive state [**17**]. For the first time, Linhan [**19**] put emphasis on the need to consider mindfulness as one of the essential components of psychotherapy. For an individual to be mindful, it is necessary to have non-judgmental awareness of his/ her emotions and thoughts occurring in the present moment. Focused attention on the present moments leads to the processing of all cognitive, physiological, and behavioral aspects of the immediate experience. Through mindfulness-based exercises, people become aware of their daily activities and the automatic function of their minds in the past and the future and through moment-by-moment awareness of thoughts, they can control their thoughts, emotions, and physical statuses and their minds become free from daily issues and automatic focus on the past and the future [**27**]. Studies have shown that mindfulness increases life quality, positive emotions and life enjoyment and decreases anger in interpersonal relationships [**19**].

The overall purpose of mindfulness training is to improve people’s compatibility with themselves, their environment, and others.

Today, the increasing rate of marital problems and conflicts has highlighted the importance of considering marital relationships more deeply. Too much conflict between married couples may lead to severe consequences and breakdown of the relationship. Consequently, considering the importance of mindfulness skills and the vital role of emotion regulation in marital relationships and their effects on the reduction of marital conflicts, the following hypotheses were tested in the present study:

1. Both mindfulness and emotion regulation training reduce marital conflicts.

2. Emotion regulation training is more effective than mindfulness training in the reduction of marital conflicts.

**Methodology**

The present evaluation was a quasi-experimental study with a pretest-posttest design and a control group. After the data collection, the obtained data were analyzed through descriptive statistics and analysis of covariance (SPSS software).

The population consisted of all the clients who referred to Moein Counseling Center in Alborz province (Spring 2014) due to marital problems. Using the simple random sampling method, 45 married people were selected as the sample and divided into two experimental groups (15 participants in each) and a control group (15 participants).

**Instruments**

**The demographic form:**this form included information such as gender, age, and educational level of the participants.

**The Marital Conflicts Questionnaire:**this 42-item questionnaire, developed by Sanaii and Barati [**3**], measured 7 dimensions of marital conflicts including the reduction of the couple’s cooperation, reduction of sexual relationships, increase of emotional reactions, increase of asking for children’s support, increase of personal relationships with their own relatives, reduction of personal relationships with the partners’ relatives and friends and separation of financial issues. Based on the standard T scores, the temporary norm of this questionnaire was calculated separately for the experimental group and men and women control groups. The minimum and maximum scores in this questionnaire were 42 and 210 respectively. Those whose raw scores were in the range of 70 to 114 (equivalent to the standard T scores between 40 and 60) had a normal marital relationship, those whose raw scores were in the range of 115 to 134 (equivalent to the T scores between 60 and 70) had more than normal marital conflicts and those whose raw scores were above 134 (equivalent to T scores above 70) were experiencing severe conflicts in their marriage and their marital relationships were extremely vulnerable.

**Procedure**

Using the simple random sampling method, 45 married people were selected as the sample and divided into two experimental groups (15 participants in each) and a control group (15 participants). At the outset, all participants answered the Marital Conflicts Questionnaire. Then, mindfulness training sessions were held for the first experimental group and emotion regulation training sessions were held for the second experimental group while the participants in the control group did not receive any training.

**Intervention methods**

**A mindfulness training:**based on the Mindfulness-Based Cognitive Therapy (MBCT) approach, eight 90-minutes sessions of mindfulness training were held for the first experimental group.

**First session:**after the pre-test, the first session started with an explanation of the importance of mindfulness training and the benefits of relaxation exercises. Then, a variety of mindfulness techniques such as focusing on breathing and some cognitive-behavioral techniques were thought. The session ended with an explanation of the homework.

**Second session:**the second session included teaching relaxation exercises for 14 muscles, physical inspection, and mindfulness in daily life, confrontation with obstacles and focus on body and face.

**Third session:**the third session included teaching relaxation exercises for 6 groups of muscles, performing yoga postures and breathing control exercises, discussing ways to confront and control thoughts and thought pattern recognition.

**Fourth session:**in the fourth session, techniques of meditation, being in the present moment and attending to misconceptions, engaging with them and choosing appropriate alternatives for them were thought.

**Fifth session:**in the fifth session, non-judgmental acceptance of experience as it actually was without trying to change it was explained.

**Sixth session:**the sixth session included mindfulness of thoughts training (i.e. noticing both positive and negative thoughts that come into the mind and then letting them go without judgment) and exercising noticing the coming of thoughts as only thoughts, not parts of the reality.

**Seventh session:**methods of self-protection and a program to prevent or deal with risk factors were explained in the seventh session.

**Eighth session:**finally, in the last session, the ways to relate the learned techniques to daily life activities were explained and a post-test was conducted.

**B. emotion regulation training** Eight 90-minutes sessions of emotion regulation training were held for the second experimental group.

**First session:**: after the pre-test, the first session started with an explanation of the importance of emotion regulation training.

**Second session:**in the second session, contents of the first session were reviewed and positive emotion awareness and its types were discussed. Then, ways to notice them were explained through examples and exercises and finally, the homework was assigned.

**Third session:**in the third session, contents of the second session were reviewed and negative emotion awareness and its types (anger, sadness, hatred, anxiety, etc.) were discussed. Then, ways to notice them were explained through examples and mental visualization.

**Fourth session:**the fourth session started with a brief review of the previous session and then ways to non-judgmentally accept positive emotions and their positive and negative consequences were thought. Homework was assigned and close relatives of the participants were questioned regarding the participants’ positive emotions swings.

**Fifth session:**: ways to non-judgmentally accept negative emotions and their positive and negative consequences were thought and homework was assigned.

**Sixth session:**two previous sessions were reviewed and then, reassessment, mental visualization, mental inhibition, and appropriate expression of positive emotions were discussed.

**Seventh session:**reassessment, mental visualization, mental inhibition, and appropriate expression of negative emotions were discussed.

**Eighth session:**in the last session, the ways to relate the learned techniques to daily life activities were explained and a post-test was conducted.

## Results

The results showed that the average ages of the participants in the three groups of mindfulness training, emotion regulation training, and control were 19.92, 33.93 and 30.73 respectively.

Regarding the participants’ educational level, 6.7% did not graduate from high school, 42.2% graduated from high school, 33.3% had BA degree and 17.8% had MA degree or higher. 68.9% of the participants were female and 31.1% were male (**Table 1**).

**Table 1:** Frequency distribution and percentage of participants based on educational level and gender in different groups

Variables		Mindfulness group		Emotion regulation group		Control group		All groups	
		Freq.	%	Freq.	%	Freq.	%	Freq.	%
Educational level	High-school	2	13.33	1	6.66	0	0	3	6.7
	High-school Diploma	6	40	6	40	7	46.66	19	42.2
	BA	4	26.66	6	40	5	33.33	15	33.3
	MA or higher	3	20	2	13.33	3	20	8	17.8
Gender	Female	10	66.66	12	80	9	60	31	68.88
	Male	5	33.33	3	20	6	40	14	31.11

**Table 2**, the pre-test/ posttest, means and standard deviations of marital conflicts are presented in the three groups.

**Table 2:** Pre-test/posttest means and standard deviations of marital conflicts in the three groups

variable	groups	Pre-test	Posttest
Marital conflicts	Mindfulness group	115.13/ 19.64	107.33/ 18.54
	Emotion regulation	120.20/ 19.92	100.47/ 15.80
	Control	118.40/ 18.68	118.60/ 16.77

To compare the effectiveness of mindfulness and emotion regulation group training in the reduction of marital conflicts, the analysis of covariance was applied in the present study. Before the analysis, it was necessary to examine its assumption. The interaction between the dispersion variable (pre-test) and the dependent variable (marital conflicts) was not significant; therefore, the data supported the assumption of homogeneity of regression slopes (p>0.05). Likewise, the parallelity and linear relationship between the variables confirmed the mentioned assumption as well. The levene’s test results concerning the variable of marital conflicts did not show any significance (F=0.861; P>0.05), confirming the homogeneity of variance in the three groups. The Kolmogorov-Smirnov test results were not significant as well (P <0.05), indicating a normal distribution of pre-test variables in all groups. Accordingly, the variables were compared between the three groups, with regard to the assumptions of covariance analysis.

Results presented in **Table 3** indicated significant differences between the experimental groups and the control group regarding the amount of marital conflicts (F=43.41; P=0.001). Therefore, the first hypothesis regarding the effectiveness of both training methods in the reduction of marital conflicts was confirmed.

**Table 3:** Covariance analysis results comparing the effectiveness of the training programs at posttest stage

Change index	Total squares	Degree of freedom	Squares mean	F value	P value	Significance level	Statistical power
Pre-test	6092.05	1	6092.05	215.18	0.001	0.89	1.00
Group membership	2458.39	2	1229.19	43.41	0.001	0.77	1.00

The results of Bonferroni test analyzing between-groups differences are presented in **Table 4**.

**Table 4:** Bonferroni post hoc test results comparing the mean scores of marital conflicts in the three groups

Groups	Means	1	2	3
Mindfulness training	107.74	-	P<0.001	P<0.002
Emotion regulation training	100.62	P<0.001	-	P<0.001
Control	118.85	P<0.002	P<0.001	-

The results presented in **Table 4** indicate significant differences between the two experimental groups and the control group regarding the amount of marital conflicts. Therefore, compared to the control group, the amount of marital conflicts was significantly reduced in the two experimental groups, confirming the first hypothesis again. Moreover, a significant difference was observed regarding the amount of marital conflicts between the two experimental groups. Therefore, the second hypothesis of “Emotion regulation training” is more effective than mindfulness training in the reduction of marital conflicts’, was confirmed.

## Discussion and conclusion

Martial conflicts significantly lack consistency between married couples, bringing about severe consequences for the families and may lead to breakdown of the marriage [**1**].

The overall objective of the present study was to compare the effectiveness of two training methods of mindfulness and emotion regulation in the reduction of marital conflicts. Comparing the pre-test and posttest scores of marital conflicts, the results indicated that the marital conflicts scores of married people in the emotion regulation group were reduced compared to the mindfulness group and scores of married people in the mindfulness group were reduced significantly compared to the control group. These findings are consistent with another study done by Denham [**11**], regarding the effectiveness of emotion regulation in the reduction of marital conflicts and the increase of intimate relationships between married couples. Results of other studies conducted by Kirby [**20**] and Abbot [**6**] regarding the impacts of irregularity of emotions on marital satisfaction were also in line with the present study findings.

Staford and Semik [**28**] found that in unhappy marriages, women showed their negative emotions frequently and men were very defensive leading to a decrease of the marital satisfaction and creation of conflicts.

Many variables directly or indirectly affect the couples’ quality of life and the frequency of conflicts between them. One of these variables is their emotions. The quality of emotions, as the most important factor in the relationships, can predict marital satisfaction.

Various emotion regulation strategies can be used in different stages of an emotional reaction. Even before the occurrence of an emotional outburst, people can feel their coming emotions; therefore, they can avoid it, limit its expression through inhibition, or change the consequences of its expression or non-expression. Thus, it seems that emotion regulation done by couples can increase their adaptive capabilities and enhance their marital satisfaction. Couples need to learn how to calm down the situation, regain their calmness, and focus on the problem again. They also have to discover how to adjust their intense emotions to different stressful situations. Hence, the attention paid to the role of emotion regulation in peoples’ coping capacity brought about the researcher’s curiosity to examine the effects of emotion regulation strategies on the reduction of marital conflicts.

In line with other studies [**16**,**19**] regarding the effectiveness of mindfulness training in reducing marital conflicts, it can be said that mindfulness meditation training is effective in the reduction of anger and anxiety in relationships, improvement of life quality [**29**] and reduction of interpersonal conflicts [**30**].

The bases of MBCT have presence of mind at any moment, preventing mental ruminations, having control over daily events, recognizing automatic thought patterns through focusing on breathing, attending to faulty recognitions and accepting confrontation with coming thoughts. Mindfulness techniques provide a way to deal with daily stressful situations. Stressors within the family are part of life and sometimes impossible to change; however, ways to deal with or respond to them can change. Since marital conflicts are significantly related to daily stresses and faulty automatic thoughts, it gives the impression that regular mindfulness exercises can create positive changes in some of the couples’ relationships and consequently enhance their marriage.

The limitations of the present study included a small number of the previous studies on the effects of mindfulness training on marital conflicts and non-equal numbers of male and female participants (the majority of participants were females).

The results of this study provided important information about the effectiveness of mindfulness and emotion regulation training in the reduction of marital conflicts. It is recommended to pay more attention to the importance of mindfulness and emotion regulation training in the improvement of interpersonal relationships, particularly relationships between married couples. It is also recommended to evaluate the durability of the effects of mindfulness and emotion regulation training on the couples’ marital relationships.
